# Metabolic changes that allow *Plasmodium falciparum* artemisinin-resistant parasites to tolerate oxidative stress

**DOI:** 10.3389/fpara.2024.1461641

**Published:** 2024-09-04

**Authors:** Alejandro David Bonive-Boscan, Héctor Acosta, Ascanio Rojas

**Affiliations:** ^1^ Centro de Cálculo Científico de la Universidad de Los Andes (CeCalCULA), Universidad de Los Andes (ULA), Mérida, Venezuela; ^2^ Max Planck Research Group Evolutionary Cell Biology, Plön, Germany; ^3^ Laboratorio de Biología y Bioquímica de Trypanosoma cruzi, Instituto de Biología Molecular y Celular de Rosario, CONICET/UNR, Rosario, Santa Fe, Argentina

**Keywords:** *P. falciparum*, metabolism, artemisinin resistance, oxidative stress, mitochondrial carrier

## Abstract

Artemisinin-based treatments (ACTs) are the first therapy currently used to treat malaria produced by *Plasmodium falciparum*. However, in recent years, increasing evidence shows that some strains of *P. falciparum* are less susceptible to ACT in the Southeast Asian region. A data reanalysis of several omics approaches currently available about parasites of *P. falciparum* that have some degree of resistance to ACT was carried out. The data used were from transcriptomics and metabolomics studies. One mitochondrial carrier of the parasite possibly involved in the mechanisms of tolerance to oxidative stress was modeled and subjected to molecular dockings with citrate and oxoglutarate. An increase in glutathione production was detected, changing the direction of the flux of metabolites in the tricarboxylic acid cycle and boosting the glucose consumed. The models of the mitochondrial carrier, called PfCOCP, show that it may be important in transporting citrate and oxoglutarate from the mitochondrial matrix to the cytosol. If so, it may allow the parasite to tolerate the oxidative stress produced by artemisinin. This *in-silico* analysis shows that *P. falciparum* may tolerate artemisinin’s oxidative stress through metabolic changes not reported before, showing the need for further experimental research on the many metabolic aspects linked to this phenotype.

## 1 Introduction

Malaria caused an estimated 229 million cases and 409,000 deaths in 2019; the majority of these were produced by *Plasmodium falciparum* (WHO, 2018). Since 2001, artemisinin-based treatments (ACTs) have been the primary drugs used against malaria produced by *P. falciparum*. Artemisinin affects the parasite in different ways: interacting with and damaging proteins, inhibiting the proteasome, and producing reactive oxygen species (ROS) (artemisinin resistance has been reviewed elsewhere (Rosenthal and Ng, 2020)). Those effects result in the death of parasites interfering with several metabolic pathways and causing systemic damage.

Despite the multilevel damage produced by the drug in 2008, some evidence suggested that the ACTs were losing their efficacy against *P. falciparum* in the Greater Mekong Subregion (GMS) in Southeast Asia (Noedl et al., 2008). Further studies show that mutations in the protein 13 of *P. falciparum* (PfKelch 13) are the main molecular markers of resistant parasites (Ariey et al., 2014), and many mutations have been identified worldwide, several associated with an increase in the number of surviving parasites in the blood (Ménard et al., 2016). However, the resistant parasites show other changes besides mutations in Pfkelch 13. Furthermore, some parasites obtained *in vitro* show an increase in tolerance to artemisinin lacking mutations in PfKelch 13 (Demas et al., 2018). One of the metabolic changes shown by these Dd2-resistant strains is an increase in the oxidative stress response (Cui et al., 2012). Also, the resistant strain Cam3.II with C580Y or R539T mutations of PfKelch13 shows higher concentrations of glutathione (GSH) and γ-glutamylcysteine, and both metabolites are directly associated with oxidative stress response (Siddiqui et al., 2017). Nowadays, a lot of evidence shows artemisinin resistance is a very complex process that may be very different depending on the genetic background (Rosenthal and Ng, 2020).

The malaria clinical manifestation occurs during the intraerythrocytic development cycle (IDC), which encompasses the asexual stages of ring, trophozoite, and schizont. During the IDC, the central carbon metabolism of *P. falciparum* is very particular; glycolysis is the main source of ATP (Roth, 1990; Jacot et al., 2016; Shivapurkar et al., 2018) since the red cell is an environment in which plenty of glucose is available. The tricarboxylic cycle (TCA), on the other hand, is not essential for producing ATP during the IDC, and the whole cycle shows high plasticity, as was proven by removing different enzymes from the pathway without causing any problem in the growth of the parasites in the IDC (Ke et al., 2015). The plasticity is even higher in artemisinin-resistant strains, based on metabolic network reconstructions (Carey et al., 2017). So far, several metabolic changes have been associated with resistance, like a reduction in hemoglobin digestion in PfKelch 13 strains (Yang et al., 2019; Birnbaum et al., 2020; Gnadig et al., 2020) or an increase in phosphatidylinositol 3-phosphate (PI3P) (Mbengue et al., 2015). Furthermore, metabolic changes have been also detected in the IDC caused by other non-artemisinin drugs (Tewari et al., 2021; Tewari et al., 2022).

An aspect that has been less studied is the mitochondrial carriers that transport metabolites involved in the TCA cycle between the mitochondrial matrix and cytosol. Among those, the *P. falciparum* mitochondrial dicarboxylate-tricarboxylate carrier (PfDTC) is the most studied (Nozawa et al., 2011). Likewise, a general approach has been used to study the transport activities of 12 *P. falciparum* mitochondrial carriers (Nozawa et al., 2020). Mitochondrial carriers may be very important to allow *P. falciparum* to change the metabolite fluxes through the matrix membrane (a.k.a inner membrane), allowing TCA cycle changes in different parasite stages. In the yeast *Saccharomyces cerevisiae*, one mitochondrial carrier (YHM2) has been involved in the oxidative stress response (Castegna et al., 2010). In this study, a probable ortholog of the yeast’s mitochondrial carrier in *P. falciparum* was identified (PlasmoDB ID PF3D7_1223800). This protein may play a similar role in *P. falciparum* contributing to the oxidative stress response, an aspect that has been previously associated with artemisinin resistance (Cui et al., 2012; Siddiqui et al., 2017) but not associated with any molecule transported from the mitochondria.

The present research will reanalyze part of the omics data about *P. falciparum* artemisinin-resistant parasites. The study proposes a metabolic model that explains a potential mechanism that allows the parasite to tolerate the oxidative stress produced by artemisinin, one of the drug’s effects that has been previously reported (Rocamora et al., 2018). The study will focus on three essential routes for the parasite: glycolysis, TCA cycle, and glutathione production. Additionally, the mitochondrial carrier PF3D7_1223800 was studied *in silico*, based on the possible importance of this carrier in the oxidative stress response induced by the drug. These approaches can show new possible therapeutic targets affecting the oxidative stress response in *P. falciparum*.

## 2 Materials and methods

### 2.1 Metabolic model of mechanisms to tolerate the oxidative stress produced by artemisinin

We used the transcriptomic profiles (Mok et al., 2015) on more than 1,000 clinical samples of *P. falciparum* in GMS and other places (Ashley et al., 2014). We chose this data set because we considered that by being obtained from so many different clinical samples, it encompasses clinically relevant changes in transcriptomics that could be associated with metabolism. These data contain 999 protein transcripts positively and negatively statistically correlated with clinical samples that show an increase in tolerance to artemisinin. We posited that differences in the transcripts’ abundance imply differences in the protein quantity. Furthermore, the metabolomic, proteomic, and transcriptomic changes using the strain Cam3.II carrying C580Y and R539T mutations in PfKelch 13 were taken into account (Siddiqui et al., 2017; Mok et al., 2021), and these mutants were also obtained from the GMS region. Other lines of evidence were not directly used, but they are discussed later in the paper, like artemisinin resistance in the Dd2 lab strain (Cui et al., 2012). The data obtained from different sources were used to construct a theoretical metabolic model of resistant strains; the metabolic pathways of *P. falciparum* were obtained from The *Malaria Parasite Metabolic Pathways* website [MPMP (Ginsburg et al., 2020)].

### 2.2 Modeling a mitochondrial carrier possibly involved in the oxidative stress response in *Plasmodium falciparum*

The putative citrate/oxoglutarate transporter (PF3D7_1223800) was renamed as the Citrate/Oxoglutarate Carrier Protein of *P. falciparum* (PfCOCP). This protein was modeled *in silico* using Phyre2 (Kelley et al., 2015) and SwissModel (Waterhouse et al., 2018) to obtain the carrier in the two different conformations that have been previously described (Ruprecht and Kunji, 2020). Phyre2 automatically uses different templates that have a good fit with the sequence supplied. SwissModel follows the same process, but the model obtained was less accurate than the one obtained with Phyre2. Finally, we used Phyre2 to obtain the carrier in the cytosolic (C) state. Phyre2 does not allow the use of a specific template. Consequently, we used SwissModel to explicitly obtain the carrier in the matrix (M) state, using the template obtained (Ruprecht et al., 2019) (PDB ID: 6GCI) of the ADP/ATP carrier from the thermotolerant fungus *Thermothelomyces thermophila* inhibited by bongkrekic acid.

The models obtained were refined using GalaxyWeb (Heo et al., 2013): twice for the model in the C-state and once for the model in the M-state. The models were refined until no improvements were found in the Rama-favored parameter, which shows how favored the protein is, based on Ramachandran’s plot (Ramachandran et al., 1963).

Molecular dockings were carried out with the refined models. This was done using two substrates obtained from the ZINC database (Irwin and Shoichet, 2005): citrate (ZINC895081) and oxoglutarate (ZINC1532519). The dockings were made using SwissDock (Grosdidier et al., 2011).

UCSF Chimera 1.13.1 (Pettersen et al., 2004) was used to visualize protein models and dockings obtained from the different servers. In addition, using the Chimera function, *FindHBond* helped us identify which residues possibly interact with citrate and oxoglutarate in the protein.

## 3 Results

### 3.1 Metabolic model of mechanisms to tolerate the oxidative stress produced by artemisinin

**Figure 1:**
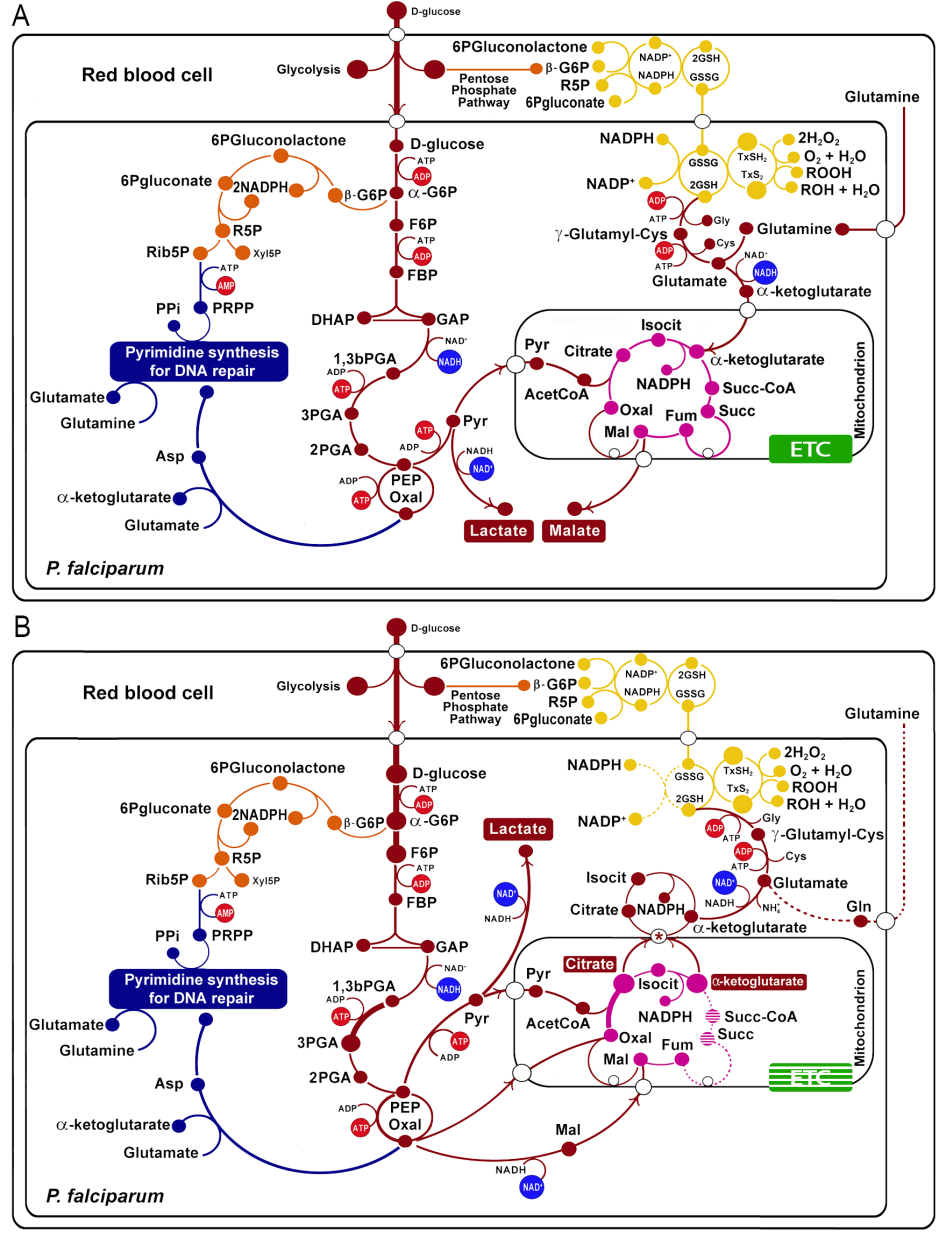
Metabolic changes in artemisinin-resistant strains of *Plasmodium falciparum* **(B)** compared with non-resistant strains **(A)**. The wide light red arrows indicate the principal flux of metabolites across the glycolysis, TCA cycle, and glutathione production. The protein transcripts positively correlated with resistance are indicated with a thick line, and those negatively correlated are indicated with a dotted line, according to (Mok et al., 2015). Glycolytic pathway in red: -G6P, alpha glucose-6 phosphate; F6P, fructose-6 phosphate; FBP, fructose 1,6-biphosphate; DHAP, dihydroxyketonephosphate; GAP, glyceraldehyde 3-phosphate; 1,3bPGA, glycerate 1,3-biphosphate; 3PGA, 3-phosphoglycerate; 2PGA, 2-phosphoglycerate; PEP, phosphoenolpyruvate. Tricarboxylic acid cycle in violet: OXA, oxalacetate; Isocit, isocitrate; Succ-CoA, succinic coenzyme A; Succ, succinate. Pentose phosphate pathway in orange: -G6P, beta glucose-6 phosphate; R5P, ribulose-5 phosphate. Purine and pyrimidine synthesis in blue: Xyl5P, xylulose-5 phosphate; Ribo5P, ribose-5 phosphate; DHA, dihydroxyacetone; PRPP, phosphoribosyl pyrophosphate. Oxidative stress branch in yellow: GSH2, glutathione reduced; G2S2; GXS2; GXSH2. Electron transport chain (ETC) in green. The asterisks indicate the PfCOCP (a citrate/oxoglutarate mitochondrial carrier).

We mainly used the transcriptomic and metabolomic data obtained from *P. falciparum* artemisinin-resistant strains (Mok et al., 2015; Siddiqui et al., 2017; Mok et al., 2021) to propose a metabolic model that shows an increase in the glycolytic path based on an increase in transcript levels of hexokinase (HK, PF3D7_0624000), phosphoglucose isomerase (PGI, PF3D7_1436000), phosphofructokinase (PFK, PF3D7_0915400), and phosphoglycerate kinase (PGK, PF3D7_0922500) in clinical samples (Mok et al., 2015). These are highlighted in **Figure 1B** (big circles).

The TCA cycle shows marked differences in the resistant strains, also based on transcript level changes. For example, citrate synthase (PF3D7_1022500, PF3D7_0609200) is upregulated, and oxoglutarate dehydrogenase (PF3D7_1320800), succinate dehydrogenase (PF3D7_1010300), and succinate CoA ligase (PF3D7_1108500) are downregulated (**Figure 1B**). These changes in the transcript levels may be related to changes in the metabolites’ influx and efflux from the mitochondrion; specifically, this can promote the efflux of oxoglutarate and the influx of malate; putatively, the transport can occur using the malate/oxoglutarate mitochondrial carrier of *P. falciparum* (PfDTC, PF3D7_0823900), which can efficiently transport these two metabolites across the membrane (Nozawa et al., 2011). For its part, citrate is overproduced and this can imply its accumulation in the mitochondria matrix. This was determined for non-resistant parasites in metabolomic analysis. Furthermore, resistant parasites show less citrate accumulation in drug presence (Mok et al., 2021), a fact that may imply that resistant parasites are better at transporting citrate to the cytosol. Citrate could be transported to the cytosol using the carrier PF3D7_1223800 (explained in the next section); this protein is a homolog of the citrate/oxoglutarate mitochondrial carrier of *S. cerevisiae* (Castegna et al., 2010).

On the other hand, metabolomic analyses were made in *P. falciparum* Cam3.II strains with R539T and C580Y mutations in PfKelch 13 (Cam3.II^R539T^ and Cam3.II^C580Y^) showing that glutathione and γ-glutamylcysteine are more abundant in these resistant strains (Siddiqui et al., 2017). This evidence was used to propose that glutathione production is overexpressed in some resistant strains (**Figure 1**). Moreover, the citrate and oxoglutarate transported to the cytosol (mentioned above) can contribute to glutathione production.

For the reduction of glutathione and thioredoxin, the parasite needs NADPH. **Figure 1** shows that diverse NADP(H) oxidoreductases carry on the reaction. Isocitrate dehydrogenase may be the primary source of NADPH in the cytosol. In fact, increased utilization of glucose by the glycolytic pathway and not by the pentose phosphate pathway (PPP), together with a decrease in the levels of transketolase transcripts (PF3D7_0610800) of PPP (Mok et al., 2015), suggests that PPP is less relevant for NADPH production in the resistant strains.

Thus far, all the changes proposed agree with an increase of oxidative stress tolerance in some resistant strains compared with sensible ones.

### 3.2 Modeling a mitochondrial carrier possibly involved in the oxidative stress response in *Plasmodium falciparum*

**Figure 2:**
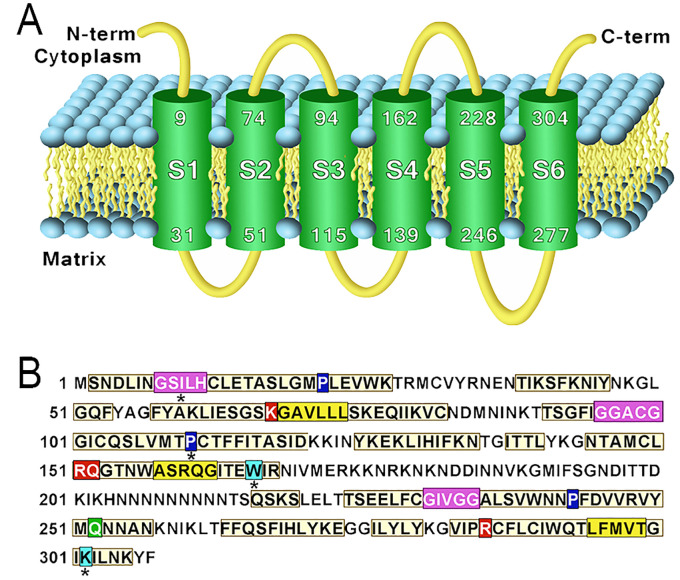
**(A)** Transmembrane regions of the model obtained using Phyre2 of *Plasmodium falciparum* mitochondrial citrate/oxoglutarate carrier (PfCOCP) in the C-state. **(B)** Sequence features found in (PfCOCP): GxxxG motif (violet), πxxxπ (yellow), Pxx[DE]xx[RK] (proline in blue), Q braces (green), Y braces (cyan), and substrate-binding sites (red). Asterisks (*) show motifs that do not match perfectly with the motifs reported in the literature.

**Figure 3:**
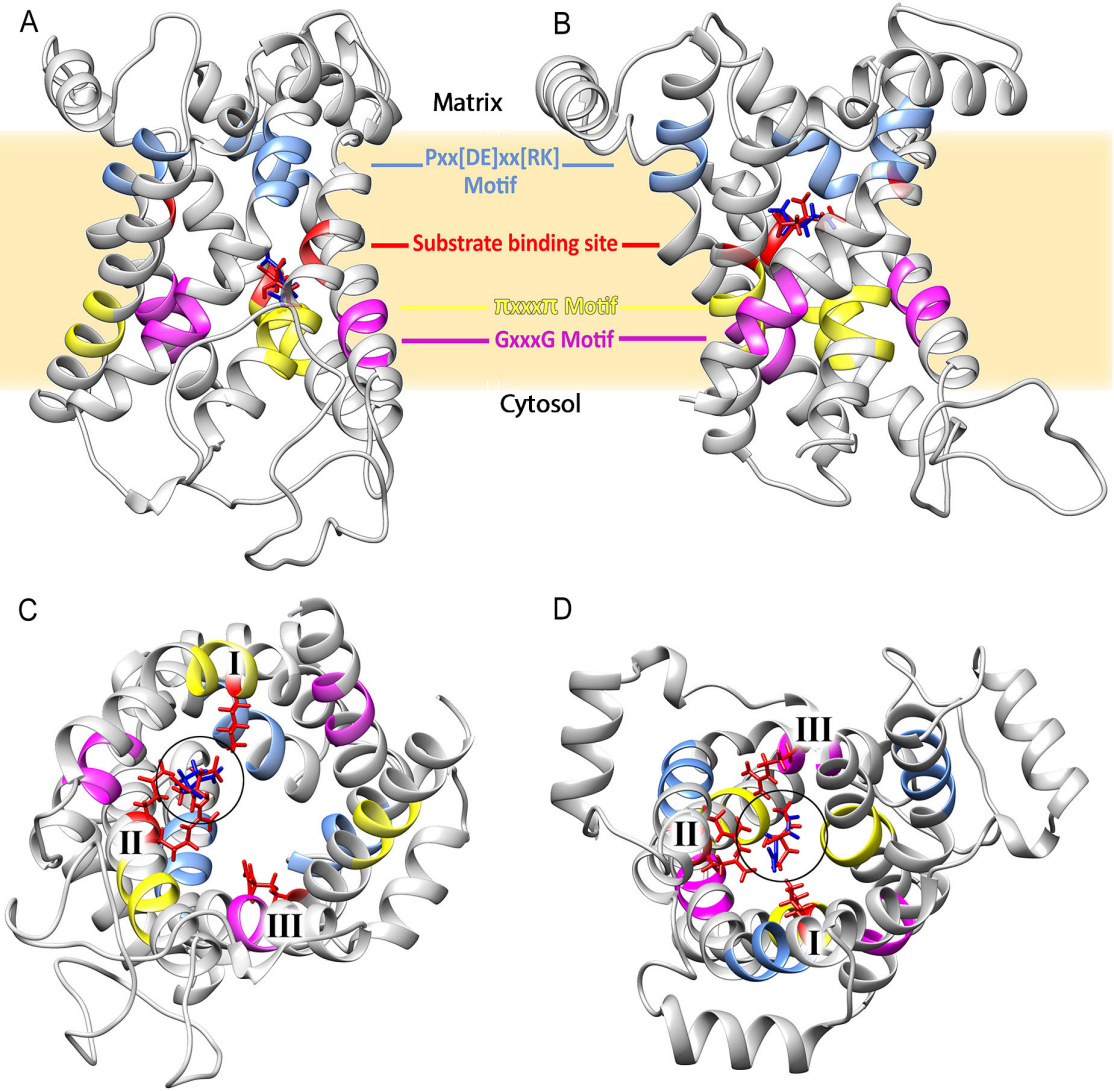
Structure of the citrate/oxoglutarate mitochondrial carrier of *Plasmodium falciparum* modeled in the cytosol **(A)** and matrix **(B)** states. Some of the sequence features are highlighted in both structures. **(C)** The protein in the C-state viewed from the cytosol. **(D)** The protein in the M-state viewed from the matrix. The chains of residues that interact with the substrates are shown in red, and the number of binding sites (I, II, and III) is shown in both cases.

The protein PfCOCP was modeled and subjected to molecular dockings with citrate and oxoglutarate. PfCOCP shows six transmembrane regions (**Figure 2A**) formed by α-helixes. These regions are going to be named H1, H2, H3, H4, H5, and H6 following other research done on these carriers [reviewed in (Ruprecht and Kunji, 2020)]. There are also three domains in the carrier’s mitochondrial matrix side that connect the different transmembrane regions, identified as H12, H34, and H56, which are also found in the works previously cited. These carriers have two different conformations: opened to the cytosol (C-state) and opened to the matrix (M-state). PfCOCP was modeled in two conformational forms, C-state and M-state (**Figure 3**), using Phyre2 and SwissModel, respectively.

We found several motifs normally identified in mitochondrial carriers (Ruprecht and Kunji, 2020) (**Figure 2B**). GxxxG was found in odd segments, namely, in H3 (**G**GAC**G**) and H5 (**G**IVG**G**), and tentatively in H1 (**G**SIL**H**). The latter agrees with the location if we highlight the H3’s and H5’s motif in the models (**Figures 3A, B**). This motif and the other regions that do not totally agree with the motif postulated in the existing literature were noted with an asterisk (*) in **Figure 2B**. πxxxπ were found in even segments: in H2 (**G**SKG**V**), H4 (**T**MWA**S**), and H6 (**T**LFM**V**). Motifs [PS]x[DE]xx[RK] were found in odd segments: H1 (**P**L**E**VW**K**), H5 (**P**F**D**VV**R**), and tentatively in H3 (**P**CTFFI). Proline residues found in [PS]x[DE]xx[RK] are important for the torsion of the transmembrane domains, and they allow mitochondrial carriers to change between both states (C and M) (Ruprecht and Kunji, 2020). One Q brace was identified in H5 (**P**F**D**VV**R**VYM**Q**) and two tentative Y braces in **Figure 2B**. These motifs have been previously described in other mitochondrial carriers (Ruprecht and Kunji, 2020). On the other hand, the [FY][DE]x[RK] motifs could not be identified, which shows that these motifs are less conserved in mitochondrial carriers (Ruprecht and Kunji, 2020).

Molecular dockings with citrate (red) and oxoglutarate (blue) are shown in **Figure 3**. Gibbs free energy (Δ*G*) values for citrate and oxoglutarate in the C-state are −18.37 kcal/mol and −24.85 kcal/mol; for the M-state, they are −21.33 kcal/mol and −31.07 kcal/mol, respectively. This suggests that PfCOCP in the M-state interacts better with both substrates. PfCOCP has three putative binding sites that interact with substrates; these are usually named I, II, and III and are located in the H2, H4, and H6 transmembrane domains of mitochondrial carriers (Robinson and Kunji, 2006). The residues that possibly interact with substrates were identified in the C- and M-states. This process allows us to propose the three sites for substrate interaction: site I in H2 (Lys 67), site II in H4 (Arg 151, Gln 152), and site III in H6 (Arg 262, **Supplementary Figure S1**). The fact that site I has a Lys, site II is composed of Arg-Gln, and site III has an Arg agrees with the substrate-binding sites of other mitochondrial carriers, namely, succinate carrier (PDB ID: FC1P) and citrate/malate carrier (TP1P), both of *S. cerevisiae* (Robinson and Kunji, 2006).

Only in the M-state of PfCOCP, the three substrate-binding sites interact with citrate or oxoglutarate; besides that, in the C-state, only sites I and II interact with substrates (**Figures 3C, D**; **Supplementary Figure S1**). These differences also suggest that PfCOCP is better at interacting with substrates in the M-state than in the C-state. The differences in Δ*G*, substrate-binding sites, and the accumulation of matrix citrate and oxoglutarate allow us to propose that PfCOCP transports citrate and oxoglutarate from the matrix to the cytosol as shown in **Figure 1**.

## 4 Discussion

The resistance of *P. falciparum* to artemisinin has been studied extensively. This resistance has been attributed to different mechanisms, which could be used by *P. falciparum* to tolerate the effects of artemisinin and may be present in the same parasites. Actually, the data used in this study are from PfKelch 13 mutant parasites, a fact that has been associated with a reduction in hemoglobin intake and heme production (Rosenthal and Ng, 2020).

This study’s metabolic pattern is consistent with an increase in oxidative stress response, which has been previously reported (Cui et al., 2012; Rocamora et al., 2018). Our model focused on three metabolic routes: glycolysis, the TCA cycle, and glutathione production.

The initial observation is the increase in the expression of the first enzymes of glycolysis: HK, PGI, and PFK, as well as PGK. Interestingly, two of these enzymes (HK and PFK) are described as enzymes with a high flux coefficient in the glycolytic kinetic model of *P. falciparum* (Penkler et al., 2015). We believe that the overexpression of these enzymes could be reflected in an increase in glucose consumption by the parasite. Additionally, overexpression of PGK could favor a higher percentage of glucose processed by the glycolytic pathway versus the PPP. Moreover, the expression levels of both glucose-6 phosphate dehydrogenase and 6-phosphogluconate dehydrogenase remain similar to those of non-resistant parasites. Likewise, in these data, transketolase is underexpressed (Mok et al., 2015). However, the route should be operative since the parasite needs the production of ribulose-5P to synthesize PRPP, which is essential for the synthesis of pyrimidines.

The use of this glucose by the glycolytic pathway will lead to phosphoenolpyruvate (PEP) formation, which is at a metabolic bifurcation point. One part of glucose is transformed into pyruvate by a pyruvate kinase (PK), providing the first net gain of ATP, while the other part of PEP can be transformed into oxaloacetate by a phosphoenolpyruvate carboxylase (PEPC) or phosphoenolpyruvate carboxykinase (PEPCK), which provides the second net ATP gain. These three enzymes (PK, PEPC, and PEPCK) do not show expression changes in transcriptomic data obtained from clinical samples (Mok et al., 2015), but in recent research with the artemisinin-resistant strains Cam3.II and Dd2 of *P. falciparum*, high levels of PEPCK transcripts have been found (Mok et al., 2021).

Another interesting change occurs in the TCA cycle where an increase in the expression of citrate synthase together with the underexpression of oxoglutarate dehydrogenase, succinate-CoA ligase, and succinate dehydrogenase would point to the functionality of only the first steps of this cycle. One consequence of this is the accumulation of citrate and/or oxoglutarate on the mitochondria, and for this, both acetyl-CoA and oxaloacetate must be supplied continuously. Thus, a metabolomic study on the artemisinin-resistant strain Cam3.II^C580Y^ showed high concentrations of citrate, which would support the previously described model (Mok et al., 2021). We speculate that the behavior of the TCA cycle in these resistant strains is similar to the one observed in *P. falciparum* parasites with deletion of the oxoglutarate dehydrogenase (ΔKDH), which would force malate to enter the mitochondria to supply oxaloacetate via MQO and oxoglutarate to exit from the organelle (Ke et al., 2015).

From the above, it follows that a constant supply of oxaloacetate for citrate synthase would require an efficient MQO. However, the decrease in the expression of components of complexes III and IV of the electron transport chain would negatively affect this supply of oxaloacetate resulting in a possible accumulation of malate. Recent metabolomic evidence suggests that malate can accumulate in Cam3.II^C580Y^ (Mok et al., 2021). Another alternative could be the direct entry of the oxaloacetate, thus avoiding the possible low functionality of MQO. In this sense, the use of the mitochondrial malate/oxoglutarate carrier of *P. falciparum* called the dicarboxylate/tricarboxylate carrier (PfDTC) (Nozawa et al., 2011) would be an alternative. This transporter proved to be efficient in transporting the oxoglutarate/malate pairs and oxoglutarate/oxaloacetate across liposome membranes, so oxaloacetate could be transported to the mitochondrial matrix and form citrate even if malate accumulates in the organelle.

Different evidence shows that the Cam3.II^R539T^, Cam3.II^C580Y^, and Dd2 strains of *P. falciparum* are obliged to constantly synthesize glutathione during infection to combat the oxidative stress produced by artemisinin (Cui et al., 2012; Siddiqui et al., 2017). In this sense, the oxoglutarate that accumulates in the mitochondria should leave and contribute to the demand for glutathione by the parasite. We also suggest that the mitochondria’s citrate could have the same fate as oxoglutarate to form glutamate in the cytosol. In this regard, during the investigation, we found that PfCOCP, a mitochondrial citrate/oxoglutarate carrier, has an increase in the level of transcripts of the mutant strain PfKelch 13 of *P. falciparum* (Mok et al., 2015). Also, we ruled out other possible fates for the citrate in the cytosol; for example, in mammals, citrate can be converted to acetyl-CoA via the ATP citrate lyase (Arnold et al., 2022), an enzyme that is not present in *P. falciparum* (Jacot et al., 2016).

We think that PfCOCP may play an important role in the transport of citrate and oxoglutarate from the matrix to the cytosol. In this context, our modeling, together with the studies of molecular couplings with citrate and oxoglutarate, shows that PfCOCP could transport citrate more efficiently from the mitochondrial matrix to the cytosol than vice versa (**Figure 3**; **Supplementary Figure S1**), a fact that agrees with the role we assigned to this transporter (**Figure 1**).

PfCOCP was proposed as a putative ortholog in *P. falciparum* of the YHM2 protein of *S. cerevisiae*, a mitochondrial citrate/oxoglutarate transporter that was associated with the response to oxidative stress in yeast (Castegna et al., 2010). PfCOCP may play this role in *P. falciparum*. It should be noted that the citrate transported to the cytosol needs two enzymes to transform into oxoglutarate: aconitase and isocitrate dehydrogenase. *Plasmodium falciparum* aconitase has been localized to the mitochondria and another subcellular region, possibly the cytosol or the digestive vacuole (Hodges et al., 2005). On the other hand, isocitrate dehydrogenase, apart from its location in the mitochondria, may have another subcellular location (Wrenger and Muller, 2003). Furthermore, in *Plasmodium knowlesi*, most of the isocitrate dehydrogenase activity has been measured in the cytosolic fraction (Sahni et al., 1992). Further research is needed to clarify whether both enzymes are in the cytosol.

In the cytosol, the oxoglutarate can be transformed into glutamate via three enzymes: two glutamate dehydrogenases (GDH1, GDH3) and the aspartate aminotransferase (AspAT). GDH1 and GDH3 use NADPH and NADH, respectively (Olszewski et al., 2009). Of those two, we think that *P. falciparum* uses GDH3 in artemisinin-resistant strains based on three factors: first, to tolerate the oxidative stress, the parasite may need NADPH for recycling glutathione and thioredoxin; second, metabolomic data obtained from resistant strains show an increase in NAD^+^ concentration (Siddiqui et al., 2017), which may be a consequence of GDH3 overuse; and third, in non-resistant parasites, GDH3 has a higher expression than GDH1 or AspAT (Bártfai et al., 2010). Notably, recent evidence obtained from proteomics shows that GDH1 and GDH3 are overexpressed in trophozoites of Cam3.II^R539T^ and Cam3.II^C580Y^, as well as high transcripts of GDH1 in the Dd2-resistant strain (Mok et al., 2021).

This glutathione synthesis is essential for the blood stages of *P. falciparum* (Patzewitz et al., 2012). Furthermore, the Cam3.II^R539T^ and Cam3.II^C580Y^ strains show significantly higher amounts of glutathione and glutamylcysteine (glutathione precursor) compared with sensible strains (Siddiqui et al., 2017). Moreover, in a recent proteomic approach, several enzymes involved in glutathione production show high values in those strains (Mok et al., 2021). In clinical samples, transcriptomic studies have shown a decrease in the expression of glutathione reductase (Mok et al., 2015), an enzyme necessary for the reduction of glutathione during oxidative stress. This was previously reported in non-resistant parasites showing that if the parasite produces more glutathione, there are less recycling of it and more efflux of oxidized glutathione to RBC (Patzewitz et al., 2012). We must also note that Dd2 *in-vitro* artemisinin-resistant strains overexpress GR (Cui et al., 2012).

This model implies that glucose may be the primary source of carbon for the TCA cycle, agreeing with the mutants obtained by Ke et al (Ke et al., 2015). Usually, glutamine is the carbon source of the TCA cycle, but the main source of glutamine is hemoglobin digestion, a process normally reduced in resistance (Rosenthal and Ng, 2020); glutamine can also be used for glutathione production.

On the other hand, several genes implicated in the mitochondrial electron transport chain (ETC) and forming ATP synthase are downregulated (Mok et al., 2015). This may indicate that those parasites are worse at producing the electrochemical gradient across the inner membrane. Interestingly, recent data obtained from a small subset of artemisinin-induced dormant *P. falciparum* parasites show that these parasites need mitochondrial matrix membrane potential to arise from dormancy (Peatey et al., 2015); the underexpression of the ETC genes may be associated with the dormancy state, previously reported for *P. falciparum* [reviewed in (Cheng et al., 2012)].

All of the above suggests that the PfKelch 13 mutant strains of *P. falciparum* would be obliged to constantly synthesize glutathione during this stage to combat the oxidative stress caused by artemisinin. The constant synthesis of glutathione would compel *P. falciparum* to produce sufficient oxoglutarate in the mitochondria from the glucose consumed.

Artemisinin resistance is a multifactorial phenomenon that can occur in different ways (Codd et al., 2011; Yang et al., 2019; Birnbaum et al., 2020). One of the limitations of the present study is that several observations from different studies were taken together, so the detection of all the changes that we mention in a single strain is still pending, despite that specific parts such as the increase in glutathione production have been properly associated to a single strain in the lab (Siddiqui et al., 2017). The omics approaches pose a very promising opportunity to discover new therapeutic targets in *P. falciparum* (Carolino and Winzeler, 2020), and the *in-silico* analysis of these data could show new targets and metabolic changes that were not pointed out before. It is important to highly consider that this study should be used as a main point to start experimental approaches to test if *P. falciparum* PfKelch 13 mutant strains can indeed increase the tolerance to oxidative stress caused by artemisinin using this metabolic model.

This *in-silico* research also suggests that PfCOCP of *P. falciparum* may have a crucial role in responding to oxidative stress caused by artemisinin. PfCOCP is thought to aid in transporting citrate and oxoglutarate from the matrix to the cytosol, where they can be converted into glutathione and NADPH. These findings unveil previously undocumented metabolic changes in artemisinin-resistant parasites and may offer new targets for treatment in *P. falciparum* strains resistant to this antimalarial drug. The *in-silico* approaches are promising in identifying potential *P. falciparum* transporters that could be effective therapeutic targets for malaria treatment, particularly considering the limited knowledge that we have of the many described transporters to date (Wunderlich, 2022). However, further investigations using *in-silico* methods are necessary, followed by *in-vitro*, or preferably *in-vivo*, studies to accurately determine the physiological function of PfCOCP, particularly under conditions of oxidative stress.

## Data Availability

The original contributions presented in the study are included in the article/**Supplementary Material**. Further inquiries can be directed to the corresponding authors.
