# Supplementary material for: Metabolic changes that allow Plasmodium falciparum artemisinin-resistant parasites to tolerate oxidative stress
Source: Front Parasitol. 2024 Sep 4;3:1461641. doi: 10.3389/fpara.2024.1461641 (PMC11731681; doi:10.3389/fpara.2024.1461641)
Supplement: Supplementary file 1 [file DataSheet1.docx]

Supplementary Material

##
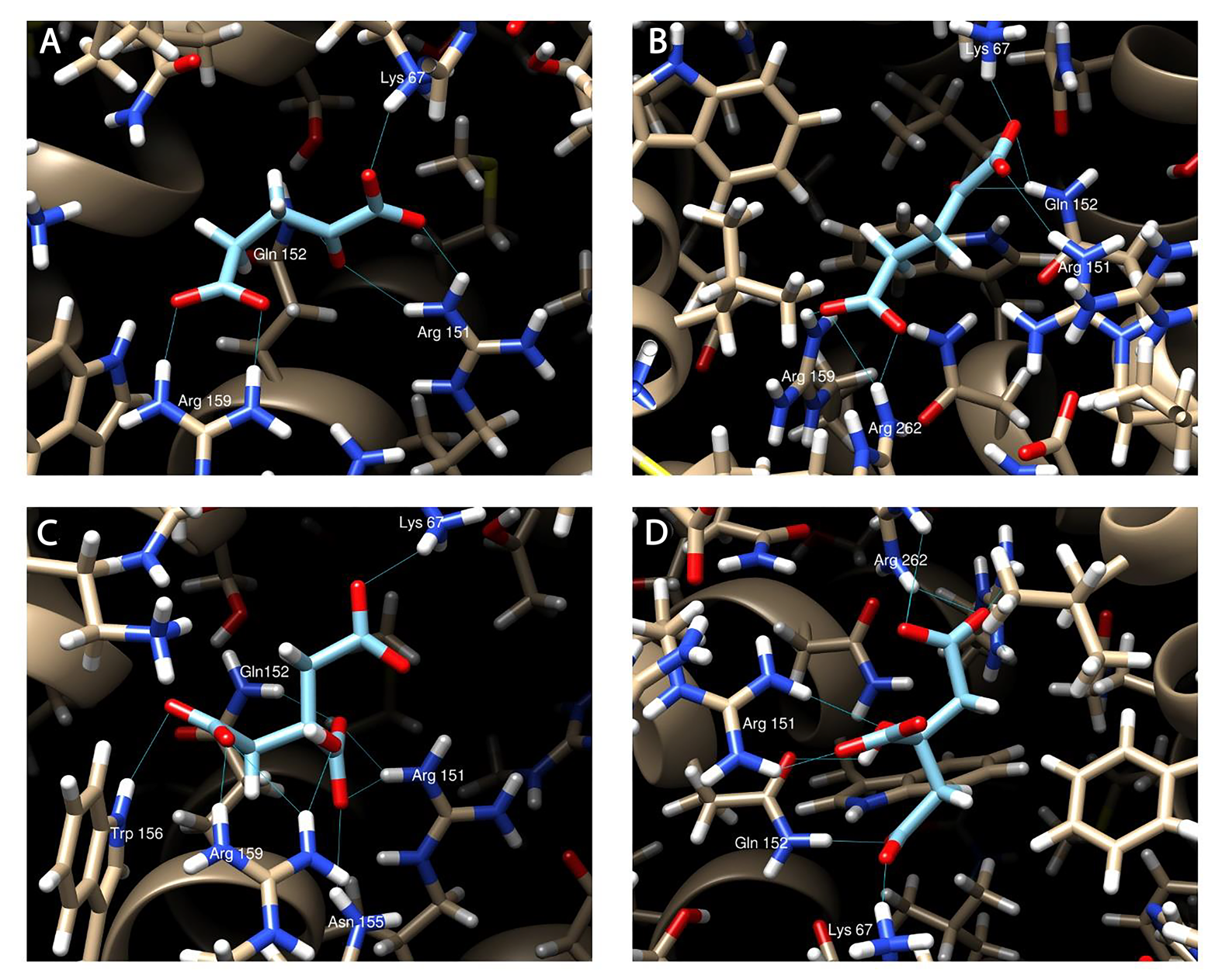
Supplementary Figures

**Supplementary Figure S1.** Molecular dockings in PfCOCP. A) and C) show oxoglutarate and citrate inside the transporter in the C state. B) and D) show oxoglutarate and citrate inside the transporter in the M state. The residues that can form hydrogen bonds (in blue) with the substrate are highlighted. The images were created using UCSF Chimera 1.13.1 (see methods).


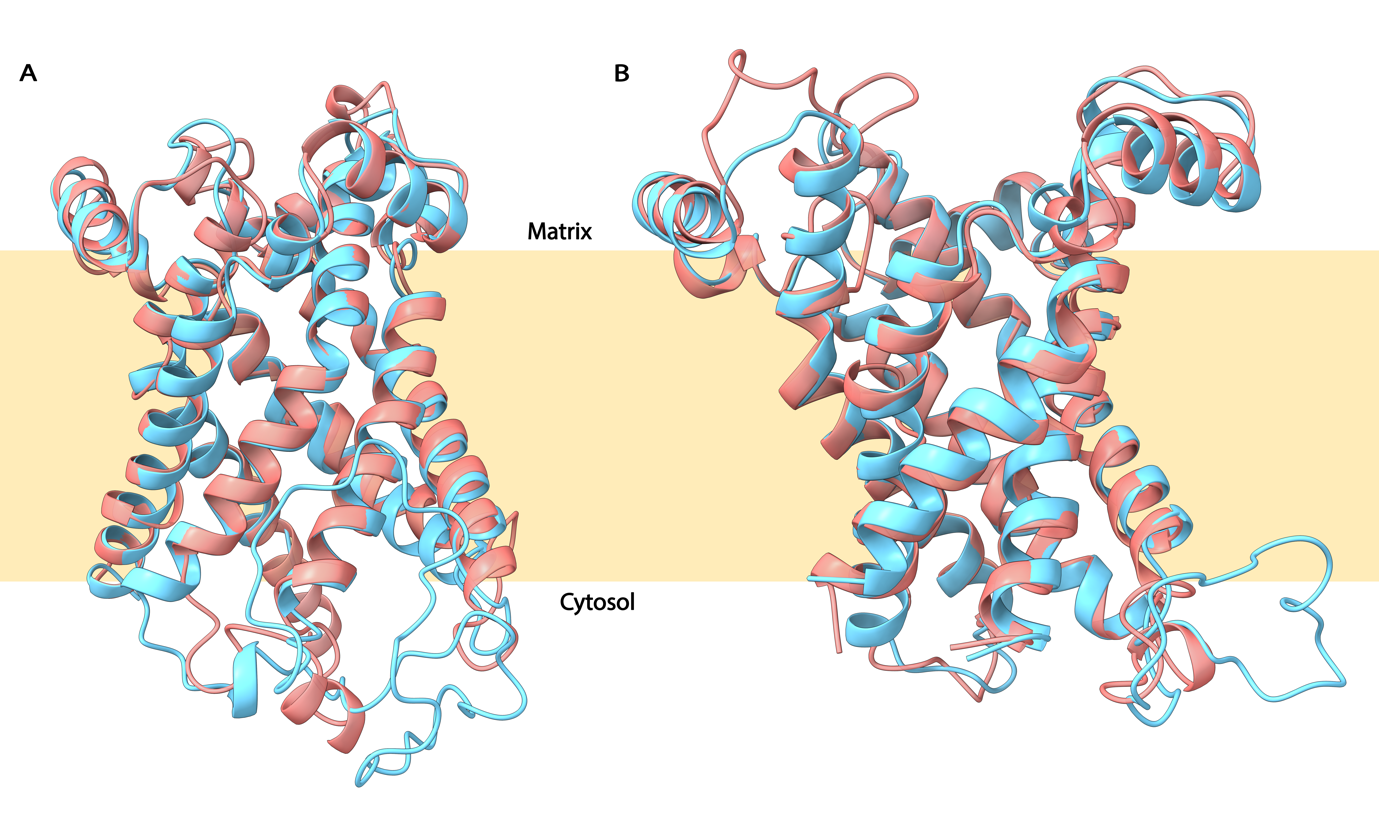


**Supplementary Figure S2.** Comparison between models of PfCOCP and YHM2 (the ortholog in *S. cerevisiae* (22)) in A) C state and B) M state. PfCOCP is highlighted in blue and YHM2 in red. YHM2 was modeled following the same methods that were used for PfCOCP (see methods).
